# Implementation Strategies for a Universal Acquisition and Tracking Channel Applied to Real GNSS Signals

**DOI:** 10.3390/s16050624

**Published:** 2016-05-02

**Authors:** Marc-Antoine Fortin, René Landry

**Affiliations:** Electrical Department, École de Technologie Supérieure (ÉTS), Montréal, QC H3C 1K3, Canada; renejr.landry@etsmtl.ca

**Keywords:** GNSS, acquisition, tracking, modulation

## Abstract

This paper presents a universal GNSS receiver channel capable of tracking any civil GNSS signal. This fundamentally differs from dedicated channels, each customized for a given signal. A mobile device could integrate fewer universal channels to harvest all available signals. This would allow securing signal availability, while minimizing power consumption and chip size, thus maximizing battery lifetime. In fact, the universal channel allows sequential acquisition and tracking of any chipping rate, carrier frequency, FDMA channel, modulation, or constellation, and is totally configurable (any integration time, any discriminator, *etc.*). It can switch from one signal to another in 1.07 ms, making it possible for the receiver to rapidly adapt to its sensed environment. All this would consume 3.5 mW/channel in an ASIC implementation, *i.e.*, with a slight overhead compared to the original GPS L1 C/A dedicated channel from which it was derived. After extensive surveys on GNSS signals and tracking channels, this paper details the implementation strategies that led to the proposed universal channel architecture. Validation is achieved using GNSS signals issued from different constellations, frequency bands, modulations and spreading code schemes. A discussion on acquisition approaches and conclusive remarks follow, which open up a new signal selection challenge, rather than satellite selection.

## 1. Introduction

Currently, most Commercially Off The Shelf (COTS) receivers available in North America only support GPS L1 C/A, while some also support GLONASS L1OF and WAAS L1 augmentation, thanks to their integration onto a single chip [1]. As new Global Navigation Satellite Systems (GNSS) are becoming available, this trend may change. Indeed, both the Chinese and Russian governments have passed laws mandating that all receivers sold in their territories be compatible with their national systems, *i.e.*, BeiDou and GLONASS, respectively [2,3]. In parallel, mobile devices (e.g., smart phones and now wearables) have also known an exponential growth.

On the other hand, higher-end receivers also support differential correction and semi-codeless tracking of the encrypted GPS P(Y) code available on L1 and L2 for improved accuracy, such as in precision farming and land-surveying [4]. Over the last decade, dedicated resources for signal-customized channels have led to receivers with more than 200 tracking channels—not to be confused with effective acquisition channels obtained through FFT-based approaches or “fast acquisition channels”—such as Javad’s [5]. These two trending markets (namely low *vs.* high end) have conflicting development paradigms: affordable battery operated *vs.* expensive and power-greedy devices.

Thanks to the modernization of GPS and GLONASS as well as the advent of Galileo and BeiDou, new signals are being broadcasted, or at least should start being transmitted shortly. These signals aim the answer the traditional GPS limitations. Indeed, higher bandwidths will help resist interferences by diluting the impact of a narrowband interference over a larger bandwidth [6]. This should also provide better positioning accuracy and resistance to multipath with a faster chipping rate [7], thus requiring a smaller correlator spacing and a higher sampling rate. Longer codes will increase signals’ cross-correlation protection and their robustness in weak signal environments. The multiplication of active satellites will increase availability, while integrity should be improved through more detailed navigation messages and deployment of new control stations, as well as new generation satellites with improved on-board clocks. This context calls for implementing new robust acquisition and tracking architectures, in a compact design, that are capable of harvesting all the potential of these new signals. Indeed, considering over 530 civil GNSS RF signal components (namely data and pilot) available worldwide, half of which being visible to any ground-based user, the importance of reducing the total complexity while maximizing global robustness and precision becomes more than desirable.

This paper novelty relies on a GNSS receiver system based on a multiplicity of the proposed universal GNSS acquisition and tracking channel based on the optimal correlator approach (*i.e.*, matched filter ([8] (Chapter 10), whose aspects are further discussed herein:

1. A dual-component (AltBOC-ready) apparatus;
2. An improved Dual Estimator code discriminator;
3. A time-multiplexing code module;
4. A secondary chip wipe-off (for longer coherent integration);
5. A configurable sub-carriers and code clocks combination module derived from a single Numerically Controlled Oscillator (NCO) master clock and
6. A sub-carrier time-multiplexing with weighted sub-carriers combination module.

### 1.1. Survey of GNSS Signals and Receiver Architectures

This section is split into three: GNSS signals, their modulation and the resulting receiver tracking channels.

#### 1.1.1. GNSS Signal Description

GNSS signals have undergone a noticeable evolution, multiplying constellations and signal definitions using new frequency bands, modulations as well as primary/secondary spreading code types, rates and periods. Global satellite-based navigation signals, with both open and restricted access on all frequency bands, are summarized in Table 1, where modulation families (detailed below) can be described as Binary or Quadrature Phase Shift Keying BPSK(q) or QPSK(q), Binary Offset Carrier BOC(p,q), Composite BOC $\text{CBOC}\left(r,\;p,\;P_{r},\pm \right)$ and Time-Multiplexed BOC $\text{TMBOC}\left(r,\;p,\;w_{r}\right)$, where:

$f_{\text{ref}}$ is the reference chipping rate, *i.e.*, 1.023 Mchip/s,
$f_{c}$ is the current chipping rate, defined as$\;q\cdot f_{\text{ref}}$,
$f_{s1}$ is the first sub-carrier rate, defined as $p\cdot f_{\text{ref}}$ and
$f_{s2}$ is the second sub-carrier rate, defined as $r\cdot f_{\text{ref}}$,
$P_{r}$ is the second sub-carrier power ratio, *i.e.*, 1/11,
$w_{r}$ is the second sub-carrier weight, in terms of an occurrence ratio, *i.e.*, 4/33.

The last parameter of CBOC refers to the sign of the second sub-carrier compared to that of the first; in CBOC, data and pilot components are in phase opposition. From Table 1, one notices that GLONASS current signals are based on Frequency Division Multiplexing Access (FDMA), while modern ones will rely on Code Division Multiplexing Access (CDMA). New GLONASS and BeiDou signals are yet to be fully publically disclosed to fill out missing details. Also, GPS L2C TMBPSK modulation is based on two alternating 511.5 kchip/s spreading codes, *i.e.*, 20 ms long CM and 1.5 s long CL, resulting in a merged stream of 1.023 Mchip/s.

Current signals and those to come have different colors; yellow cells identify memory codes.

The Mean Time To Acquire (MTTA) in Table 1 is computed per the worst case scenario represented by Equation (1) [9]:

(1) $$MTTA=\frac{\left(2-P_{D}\right)\left(1+K\cdot P_{FA}\right)}{2P_{D}}b\cdot M\cdot T_{P}$$

where:

$P_{D}$ is the detection probability (assumed at 0.995)
$P_{FA}$ is the false alarm probability, *i.e.*, a false positive (assumed at 0.001)
K is the false alarm weight (assumed at 2)
b is the number of search cells (combining code with 0.5 chip resolution and ±5 kHz Doppler span)
M is the non-coherent integration count (assumed at 1)
$T_{P}$ is the pre-integration time (assumed to match primary code period)

In the case of the GPS L1 C/A signal, there could be 2046 code bins and 15 frequency bins, each spaced by 667 Hz assuming 1 ms integration and covering a ±5 kHz Doppler span. This sequential acquisition scheme would result in a total of b=30,690 search cells. In Table 1, MTTA is applied to both primary and secondary spreading codes, although secondary code could be extrapolated from the message time stamp instead of being searched. To sum up the review of GNSS signals, there are 291 civil GNSS RF signal components (*i.e.*, considering both data and pilot components) currently available worldwide: six signal components on 32 GPS satellites + 2×24 for GLONASS + 6×6 for Galileo + 3×5 for BeiDou, as listed in Table 1, half of those could be visible to any ground user. Hence, in order to harvest this signal power to maximize global robustness and precision, reducing receivers’ total complexity and reusing as many resources as possible becomes more than desirable. The number of signal components available will increase to more than 530 as the new satellite constellations are deployed, on top of the local and augmentation signals...

#### 1.1.2. GNSS Signals Modulations

One of the most complex modulations involves two sub-carriers—namely SC1 & SC2—in the Multiplexed BOC (MBOC) scheme, which is defined as a spectrum (*G*) involving BOC(1,1) and BOC(6,1) in a 10 to 1 power ratio:

(2) $$G_{\text{MBOC}}\left(f\right)=\frac{10}{11}G_{\text{BOC}\left(1,\;1\right)}\left(f\right)+\frac{1}{11}G_{\text{BOC}\left(6,\;1\right)}\left(f\right)$$

This power ratio allows for a smaller bandwidth to be processed in low-end receivers, while still achieving lock. MBOC is found in two different implementations. In Galileo, the Composite BOC (CBOC) data and pilot signal components first sub-carriers end up in counter-phase, while their second carriers remain in phase, after being combined $s_{E1}=s_{E1-B}-s_{E1-C}$ [10], with:

(3) $$s_{E1-B}\left(t\right)=\;PC_{E1-B}\left(t\right)\;\cdot d\left(t\right)\cdot \text{CBOC}\left(6,1,1/11,+\right)\left(t\right)\;s_{E1-C}\left(t\right)=\;PC_{E1-C}\left(t\right)\;\cdot SC_{E1-C}\left(t\right)\cdot \text{CBOC}\left(6,1,1/11,-\right)\left(t\right)$$

with the Primary Code (*PC*), Secondary Code (*SC*), navigation data (*d*), and:

(4) CBOC(6,1,1/11,±)(t)=1011BOC(1,1)(t)±111BOC(6,1)(t)

In GPS, the Time-Multiplexed BOC (TMBOC) only involves the second sub-carrier in the pilot signal component, enabled four times within a 33-chip long pre-determined sequence [11]:

(5) $$s_{L1C_{D}}\left(t\right)=\;PC_{L1C_{D}}\left(t\right)\;\cdot d\left(t\right)\cdot \text{BOC}\left(1,1\right)\left(t\right)\;s_{L1C_{P}}\left(t\right)=\;\sqrt{3}PC_{L1C_{P}}\left(t\right)\;\cdot SC_{L1C_{P}}\left(t\right)\cdot \text{TMBOC}\left(6,1,\;4/33\right)\left(t\right)$$

with:

(6) $$\text{TMBOC}\left(6,1,\;4/33\right)\left(t\right)=\alpha \left(t\right)\cdot \text{BOC}\left(1,1\right)\left(t\right)+\beta \left(t\right)\cdot \text{BOC}\left(6,1\right)\left(t\right)\;\alpha \left(t\right)=\{\begin{array}{c} 1,\;t\in 29/33\\ 0,\;t\in 4/33 \end{array}\;and\;\beta \left(t\right)=\{\begin{array}{c} 0,\;t\in 29/33\\ 1,\;t\in 4/33 \end{array}$$

For all open signals, sub-carriers are in phase with the chip transitions, *i.e.*, sine BOC (sBOC). The only signals using cosine BOC (cBOC), with chip and sub-carriers in quadraphase, are Galileo E1A and E6A, which are respectively regulated and commercial services.

For Galileo E5, the Alternate BOC (AltBOC) modulation offers two QPSK channels symmetrically offset from a common center frequency, resulting in a 51.150 MHz wide receiver reference bandwidth encompassing the 20.460 MHz large main lobe of both open E5-A and Safety of Life (SoL) E5-B signals [10]. Processing this signal as a whole would require an even higher sampling rate (as per Nyquist), especially if secondary lobes are considered. In fact, the combination of these two signals into a single transmission primarily serves the goal of optimizing the usage of the on-board satellite power amplifier through the constant complex power envelope of a PSK-8 signal, yet with improved receiver multipath performances [12]. Anyhow, each signal component requires independent correlators. In the context of this paper, E5A and E5B are processed independently as QPSK (10) signals.

#### 1.1.3. BOC-Ready Tracking Channels

The main complication introduced by the new signals is the ambiguous BOC Auto-Correlation Function (ACF). Indeed, the squared ACF introduces the possibility of tracking any 2n−1 peaks separated by the sub-carrier half period $T_{s}$, with the sub-carrier to chipping rates doubled ratio $n=2\cdot f_{s}/f_{c}\;$. In the simplest case of BOC(1,1), there are two side peaks, whose tracking would induce a Pseudo-Range (PR) error of ~150 m. Hence, BOC ACF ambiguous tracking [13], requires adapted tracking approaches, some of which are categorized in Table 2.

## 2. Materials and Methods

### 2.1. Universal GNSS Channel Design Decisions

**Figure 1:**
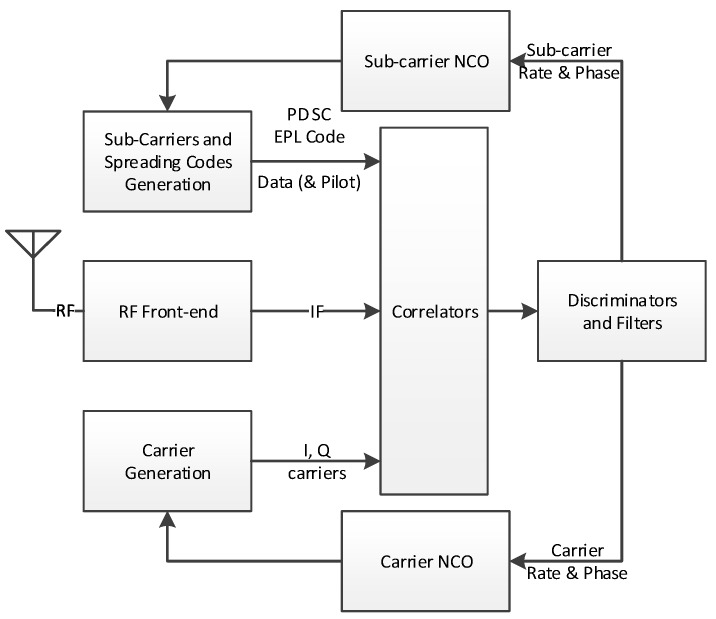
Tracking Channel Simplified Architecture.

For a universal channel [32,33] to successfully address any signal particularity identified above, some design decisions had to be made to achieve the lowest possible design complexity. The following paragraphs detail different channel architecture aspects (*cf.* Figure 1), *i.e.*,:

1. IF to Baseband Down-conversion and Carrier (including FDMA) Wipe-off module
2. Sub-carriers and Spreading Codes Wipe-off module
3. Spreading Codes (including Time Multiplexing) Generation module
4. Correlation module
5. Data & Pilot components merging
6. Discriminator and Filters

An overall resource and power assessment is then presented.

#### 2.1.1. Carrier

Assuming that all RF signals are taken down to a common Intermediate Frequency (IF), it then becomes possible to track any GNSS signal with the proposed universal channel. In order to accommodate most GNSS signals, a 30 MHz processed bandwidth appears to be a good compromise. This imposes a 60 MHz real sampling frequency and a 15 MHz Intermediate Frequency (IF), common to all RF bands. This architecture is thus compliant with all open signals.

A local carrier complex oscillator (namely a pair of sinusoidal 64-point waveforms in phase quadrature and encoded on 4 bits) is used to convert the IF signal down to baseband. Furthermore, in order to preserve a low architecture complexity, a signed multiplication optimization is proposed: Y bits×Z bits=(Y+Z−1) bits. This is true only if the minimal twos complement value is never used on both operands, e.g., 0b0000×0b0000 would not be permitted in such 4-bit multiplications.

The flexibility offered by this frequency down-conversion allows simplifying the RF front-end. Indeed, a common RF front-end could be used to manage signals on carriers nearby one another, such as:

- Galileo E5B and Beidou B2-I (and eventually B2b) on 1207.14 MHz, as well as 1202.025 MHz for GLONASS L3 signals.
- Beidou B3 on 1268.52 MHz as well as Galileo (and QZSS) E6 signal on 1278.75 MHz:
  ○ In order to preserve both signals bandwidth integrity, the RF front-end would take 1273,635 MHz down to IF. Assuming IF = 15 MHz, Beidou B3 would manage 20 MHz for its QPSK (10) signal as well as 10 MHz for the Galileo E6B/C BPSK (5) signals.
  ○ This simplified approach could only process half of Galileo E6A BOC(10,5) and Beidou B3-Ad/Ap BOC(15,2.5) signals, considering the current 30 MHz bandwidth.
- Beidou B1-1 on 1561.098 and B1-2 on 1589.74 MHz around GPS L1 (and others) on 1575.42 MHz:
  ○ An alternate approach would be to implement a 14.322 MHz sub-carrier, thus dealing with both Beidou signals as sBOC(14,2), just as with Galileo E1A cBOC(15,2.5), but with a slight sensitivity loss caused by superposing these two signals, each having their spreading code providing >20 dB isolation.

More importantly, dealing with the several frequency channels of the GLONASS FDMA scheme requires a Numerically Controlled Oscillator (NCO) frequency span over several MHz, *i.e.*, [−7, 6]⋅0.5625=7.3125 MHz for L1OF and equivalently 5.6875 MHz for L2OF:

(7) $$f_{L1OF}=1602+0.5625\cdot \left[-7,\;+6\right]\text{ MHz }\;f_{L2OF}=1246+0.4375\cdot \left[-7,\;+6\right]\text{ MHz}$$

This NCO span represents a large increase compared to the traditional ±10 kHz required for Doppler removal for a high-dynamics receiver.

#### 2.1.2. Sub-Carriers

As seen in Table 1, signal modulations involve up to two sub-carriers combined in different phase relations. In fact, a phase-controlled sub-carriers generation module based on a single NCO makes up a universal channel. This NCO is used to derive up to two slower periodic signals from a third one (*i.e.*, SC2); the slowest signal being used to dictate the chipping rate of the primary spreading code. By doing so, an NCO phase ambiguity issue arose, which was overcome with the introduction of a SC2 period counter used in the navigation solution algorithm.

**Figure 2:**
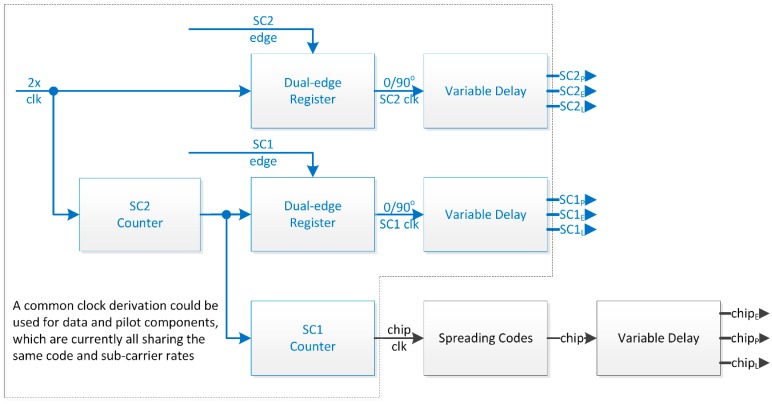
Sub-Carriers and Spreading Codes Module (BPSK *vs.* MBOC).

To properly deal with signals characterized by a quarter of a cycle phase shift between chip transition and carrier rising edge (*i.e.*, cBOC), a minimalistic approach requires a source clock with twice the required rate and a dual-edge register, as depicted in Figure 2. This approach would equally apply to Galileo E1A signal with cBOC(15,2.5), where a sub-carrier six times that of the spreading code rate, both clock signals being in phase quadrature.

Another requirement brought up by the sub-carriers is their respective weight in time. Indeed, the TMBOC pilot component requires the ability to null (*i.e.*, switch off) sub-carriers in time. To be future-compliant with any periodicity length, applied on any sub-carrier, a single 16 kbit RAM block is used, achieving a maximum periodicity of 512 addresses × 32 bits/(2 components × 2 sub-carriers) = 4096. To use it efficiently, the RAM block is configured as a dual port RAM, written from the 32-bit data bus until the RAM is filled up, but only four bits are read per address to accommodate data and pilot components at once.

**Figure 3:**
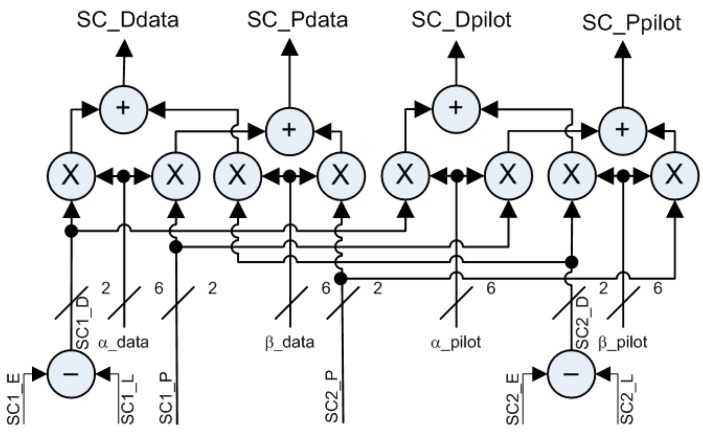
MBOC Sub-Carriers Multi-Bit Simplification and Combination of both Data and Pilot Components (Differential = Early − Late; Prompt)

Furthermore, CBOC and TMBOC impose different sub-carrier amplitudes. Pursuing a matched filter approach, the replica should mimic the targeted signal as much as possible. The resulting weighing factors α for sub-carrier SC1 and β for SC2 must carry the following values: α∈[1, 0.95, 0] and β∈[1, ±0.30, 0]. The signed resolution requires a total of 6 signed bits to induce a representative ratio between one another: $\frac{\beta }{\alpha }=\frac{0.30}{0.95}=\frac{6/32}{19/32}$ with α+β=25/32, introducing a potential scaling loss. These 6-bit coefficients may be updated at every chip in this simple TMBOC implementation, as depicted in Figure 3. For example, during the sequential acquisition process, four steps are followed:

1. For each component, both 1-bit square sub-carriers are delayed to obtain Early (E), Prompt (P) and Late (L) replicas; the correlator spacing is set to $\pm \;T_{s}/4$ with the fastest sub-carrier period $T_{s}$.
2. Prompt and Differential (D = E − L) are obtained on 2 bits for each sub-carrier.
3. P & D replicas are scaled to their pre-defined constant weight through a mapping function or Look-Up Table (LUT).
4. For each component, the two scaled sub-carriers are summed.

#### 2.1.3. Spreading Codes

As seen in Table 1, codes have different lengths and generation methods. Since all signals have their own primary (and secondary) code generation method, a universal channel would need to support them all. Linear Feedback Shift Register (LFSR) logic is definitely the best approach in the case of a dedicated signal channel. However, duplicating such resources customized for every signal becomes a burden: one channel can only track one signal at a time, resulting in many idle resources. Furthermore, considering this highly dynamic field, one may want to plan ahead. Indeed, a pre-computed memory code approach not only applies to all currently defined signals, but also allows for an easy, over-the-air, update link whenever a new Signal In Space (SIS) Interface Specification (IS) is released.

In recent GNSS signals, longer code periods also reduce the transit time integer ambiguity; the transit time for GPS satellites on L1 varies from about 66 ms (at zenith) to 80 ms (at horizon) [34]. Hence, the longer the code duration, the smaller the resulting ambiguity becomes. To further improve on this, secondary codes are laid over the primary ones, artificially making them longer (while improving the inter-correlation protection). To account for the secondary code, whose length may vary from 4 to 1800 chips, the memory codes approach is once again adopted. Another side effect of these secondary codes is the basic integration time period: they constrain the coherent integration time to the primary code period, which in turn, limits the correlation gain achieved during acquisition (at early acquisition stages, while the secondary code is still unknown).

The only civil code for which the memory code approach is not suited is GPS L2CL. Indeed, CL is 767,250-chip long, which would impose a much too high upper bound on the size of the memory dedicated to each channel, especially if we consider two such memory blocks (one for each component). A more realistic memory block size is 16 kbit (a standard size for the Virtex4 [35], on which the proposed universal channel is implemented), which is greater than 10,230—the second longest code, found on the L5, E5 and B3 signals. Hence, this requirement imposes two 16 kbit RAM blocks and a 27-register long LFSR as the minimum resources for each universal channel.

**Figure 4:**
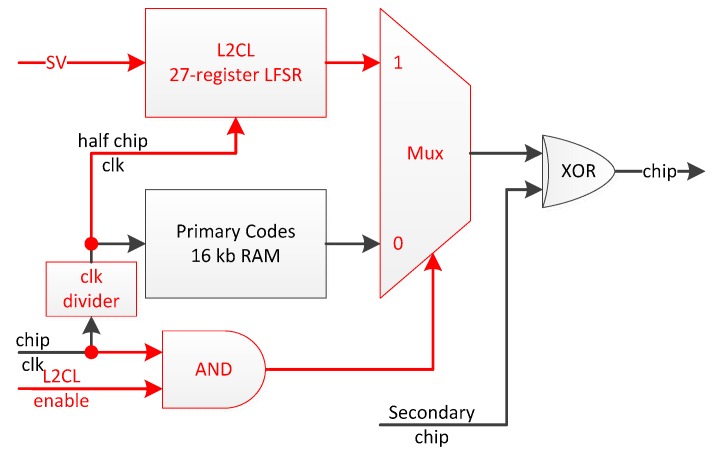
Spreading Codes Module (BPSK *vs.* TMBPSK Overhead).

More importantly, the GPS L2C signal introduces an additional particularity, *i.e.*, the time multiplexing of two spreading codes of different lengths. The resulting merged code has twice the chipping rate compared to that of their individual sequences L2CM and L2CL, as seen in Figure 4.

The 1.5 s long L2CL code cannot be acquired directly at cold start. Nevertheless, its chip offset can be predicted from the satellite clock timestamp decoded through L2CM or inferred from another signal from the same satellite. A full (L2CM & L2CL) integration may then occur, harvesting twice as much signal power compared to only L2CM during the acquisition phase.

#### 2.1.4. Correlation

**Figure 5:**
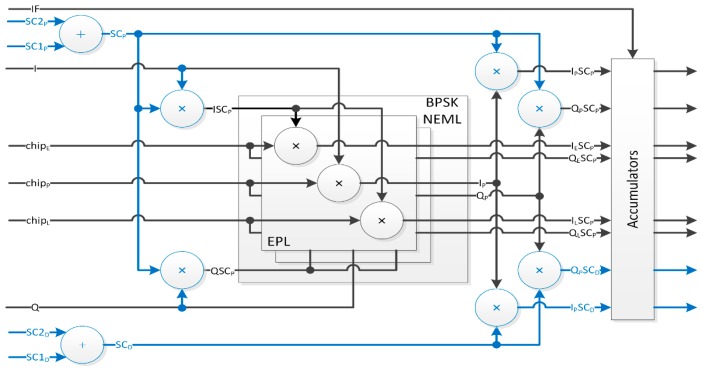
Single-Component Products and Correlation (BPSK *vs.* MBOC Overhead).

In order to provide the feedback to the carrier and code NCOs, several feedback signals are required to compute the error to be compensated for. For the code, a Non-coherent Early Minus Late (NEML) discriminator requires three correlators, *i.e.*, E, P and L code replicas on both the phase (I) and quadrature (Q) branches as illustrated in Figure 5. With the current implementation based on 4 16-bit addressable registers (as opposed to dedicated RAM blocks with improved delay resolution), the different code replica offsets may belong to $P\pm 32/f_{S}$ samples, thus achieving a correlator spacing Δ=±δ slightly larger than ±½ chip for a 1.023 Mchip/s spreading code. The resulting 6 correlators are deployed for both components of a signal.

To reduce the correlator number, the Delay Lock Loop (DLL) discriminator could only involve in-phase (carrier and eventually sub-carrier phases) measurements, thus requiring a lock on the Phase Lock Loop (PLL) (and eventually Sub-carrier Lock Loop or SLL). Such a coherent approach may not be as robust as its non-coherent equivalent [36].

Hodgart, Blunt and Unwin [37] specify that an SLL provides more precise (due to higher rate), but ambiguous (periodic clock signal) measurements compared to the DLL based on the primary code Pseudo-Random Noise (PRN). Both these estimates may be combined as:

(8) $${\hat{\tau }}^{+}={\hat{\tau }}^{*}+round\left(\frac{\hat{\tau }-{\hat{\tau }}^{*}}{T_{s}}\right)T_{s}$$

where:

${\hat{\tau }}^{+}$ is the combined delay estimate;
${\hat{\tau }}^{*}$ is the sub-carrier delay estimate;
${\hat{\tau }}^{}$ is the code delay estimate;
$T_{s}$ is the sub-carrier half-period.

To keep the correlator count as low as possible, the sub-carriers are weighted (α,β) and summed, *i.e.*, SC2 + SC1, prior correlation, avoiding an extra loop. Also, only Prompt (P) and Differential (D = E − L) instances are used to implement the NEML sub-carriers discriminator. Note that combining the sub-carriers also simplifies the discriminator, which then becomes identical as the Dual Estimator (DE), rather than the Triple Estimator (TE) extension for MBOC [25], with the same performances.

Having higher chipping rates requires greater accumulation registers. Multiplication and accumulation are performed through a DSP48 slices available in the Xilinx XC4VSX55-10FF1148 Field Programmable Gate Array (FPGA). Hence, the number of bits for these operations is not critical, as long as it remains below $48-\log_{2}\left(60,000\right)\approx 32$, assuming the integration of 60,000 samples in 1 ms.

Coherent integration provides better post-correlation Signal to Noise Ratios (SNR) than non-coherent ones, where navigation bit (or secondary chip) removal introduces squaring losses [38]. The navigation data period limits the coherent integration time, thus imposing a lower limit on the sensitivity of an unaided, stand-alone receiver.

#### 2.1.5. Data & Pilot Components Merging

Most new and modernized signals have two components combined in (counter-) phase or in phase quadrature, such as Galileo E1 B&C and GPS L5 I&Q, respectively. In order to deal with them, a special design choice has to be made: either (1) each component is dealt with in a separate channel, whose correlation products are properly dealt with through a common (or distinct) discriminator; or (2) both components are integrated into one dual-component universal channel. Although more flexible, the first case would not allow for the HW reduction of the following shared resources (in the authors’ opinion, when applied to the proposed architecture):

1. Memory codes address and control logic.
2. Carrier and sub-carrier NCOs direct and derived clock signals.
3. Sub-Carrier generation of Prompt (P) and Differential (D) shared by both signal components as the data and pilot chipping rates are always equal in the publically disclosed signals.
4. Sine and cosine LUTs and carrier multipliers leading to the I and Q branches.
5. 27-stage LFSR L2CL code generation implemented only once per dual component channel (*i.e.*, implementing more than 32 universal channels would waste even more resources as there should not be more than 32 L2CL codes being broadcast by the current GPS constellation).

Thus, several architectures are possible, depending on the receiver performance *vs.* cost desired ratio. Dual-component channels allow maximizing the harvested signal power, whereas single-component architectures only allow one of the following:

- Acquire and track the data component only, ignoring the pilot component available power.
- Acquire pilot component with a longer integration time for greater sensitivity and then transfer to data component tracking to extract the navigation message.
- Acquire and track both pilot and data components in independent channels.

With the dual-component channel resources available, a faster sequential acquisition also becomes possible by splitting the search space into two sets of chip offsets:

1. Dual-code delay search makes primary code acquisition two times faster and;
2. Once synchronized onto the primary code, a dual secondary chip estimation (*i.e.*, either the secondary chip changes or not) allows for an integration time over twice the primary code period by using the best of these two integration outputs.

In order to minimize power consumption in mobile devices, the pilot-related components may become idle during single-component signal tracking.

#### 2.1.6. Discriminator and Filter

In a multi-signal receiver, the phase relationship from one signal to another may not be cancelled out as part of a common timing error and must thus be specifically accounted for. Similarly, dual-component signals are bound by their phase relationship. With a standard definition where the quadra-phase component leads the in-phase one, we have:

(9) s=sin(x)+j⋅cos(x)

That is to say, an in-phase (e.g., sin) signal (such as GPS L1CI) may use the I and Q correlator values, while a signal in phase quadrature (e.g., cos) with its RF carrier (such as GPS L1 C/A) should use –Q and I. In the current implementation, the discriminators are programmed into the embedded MicroBlaze controller, thus allowing for great flexibility. Basically, any coherent and/or non-coherent discriminator could be used based on the signal characteristics; this is simpler than generating sinusoidal waveforms with different phases.

**Figure 6:**
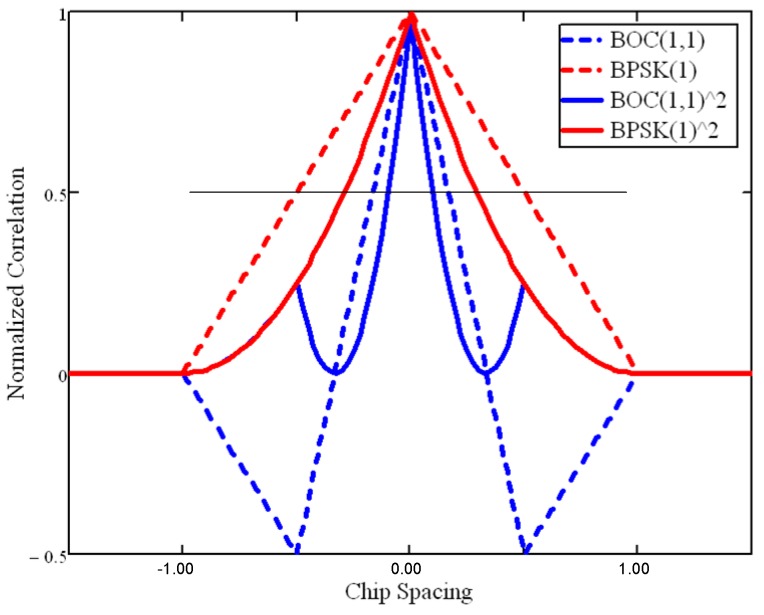
Infinite Bandwidth BPSK (1) and BOC(1,1) − n = 2 − Coherent and Non-Coherent Normalized Correlation Functions.

**Figure 7:**
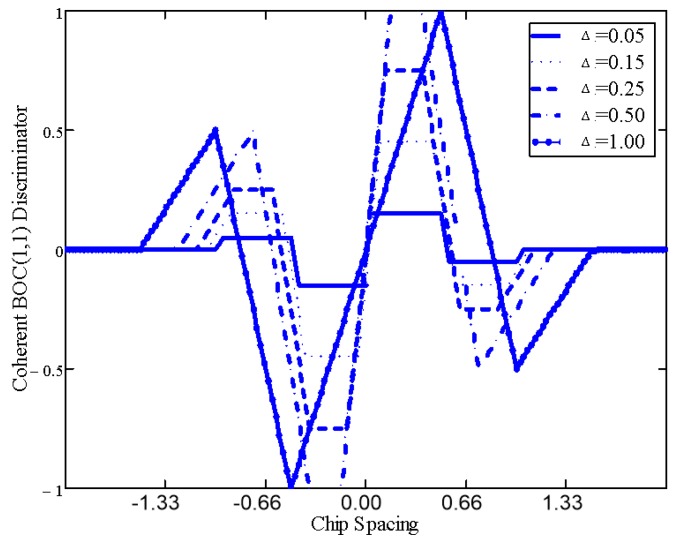
Effect of Correlator Spacing (Δ (chip)) on a BOC(1,1) Coherent EML Discriminator (assuming an infinite front-end bandwidth).

More precisely, considering the infinite bandwidth signal auto-correlation function, Figure 6 shows that the BOC main peak has a slope of ±1.5n and a correlation main peak width of $\pm \frac{1}{n}$ chip, with the BOC modulation ratio $n=2\cdot \frac{f_{s}}{f_{c}}=2\cdot \frac{p}{q}$. However, the squaring involved in non-coherent correlation steepens the peak slopes: which can be approximated by ±2n between the correlation peak and the zero-amplitude level, separated by approximately $\pm \frac{1}{2n}$ chip. The coherent correlator spacing should not extend beyond $\pm \frac{1}{n}$ chip, above which an inversion of the EML discriminator S-curve in Figure 7 could compromise the DLL behavior (*i.e.*, it would amplify the error) [39]. Each one of the 2(n−1) side peaks in the squared BOC correlation function leads to a potential false-lock (*i.e.*, a biased discriminator output) as a result of as many side S-curves.

Also, a rule of thumb imposes, neglecting dynamic stress error ([8] (Chapter 5):

(10) $$3\cdot \sigma_{\tau_{EMLP}}^{}<\delta$$

The normalized correlation function R(τ±δ) is estimated by its main peak positive and negative slopes: {1+m(τ−δ)} and {1−m(τ−δ)} with EML correlators spaced by ±δ chip and a chip code delay error $\left|\tau \right|<\frac{1}{2n}-\delta$. The EML tracking architectures for BOC, should offer a code tracking improvement of m over BPSK.

In non-coherent discriminators, $C/N_{0}$ squaring losses are due to doubled random noise, while the ±1 data is wiped off. Non-coherent processing would typically be 3 dB less sensitive than coherent processing for a given duration, although it allows for much longer integration periods, thus achieving a better overall sensitivity. This squaring loss was isolated in square brackets in the code noise jitter equations below. Hence, in non-coherent discriminators, the associated code noise may have a larger variance while preserving the same null mean. It is well known that code phase jitter performances depend on the slope of the discriminator curve (*i.e.*, better performances for steeper slopes). In fact, the code phase 1 -σ error (m) derived from the non-coherent Early Minus Late Power (EMLP) code discriminator closed loop noise variance (squared chip periods) are defined as (extended from [12,40] to BOC derived modulations):

(11) $$\sigma_{\tau }≅c\cdot T_{c}\cdot \sqrt{\sigma_{\tau_{EMLP}}^{2}}$$

(12) στEMLP2≅{BL(1−0.5BLTP)2⋅CN0⋅[1+ 4CN0⋅TP⋅(2−m⋅Δ)]⋅{Δm}, π≤Δ⋅bBL(1−0.5BLTP)2⋅CN0⋅[1+ 4CN0⋅TP⋅(2−m⋅Δ)]⋅{1b+bπ−1⋅(Δm−1b)2}, 1<Δ⋅b<πBL(1−0.5BLTP)2⋅CN0⋅[1+ 2CN0⋅TP]⋅{1b}, Δ⋅b≤1

where:

c is the speed of light (m/s);
$T_{c}$ is the chip period, the inverse of the chipping rate $f_{c}$;
$B_{L}$ is the unilateral noise equivalent bandwidth of the code tracking loop, a.k.a. one-sided equivalent rectangular bandwidth, with the time frame of interest $\epsilon \;\left[1/B_{L},T_{obs}\right]$;
$T_{P}$ is the pre-integration time (s);
τ is the signal *vs.* replica misalignment (chip);
Δ is the early-late correlator spacing (chip), *i.e*, 2⋅δ;
δ is the early to prompt and prompt to late correlator spacing (chip);
CN0 is the Carrier power to Noise density ratio (dB-Hz);
m is the slope of the correlation function;
b is the normalized receiver front-end complex bandwidth $\left(\beta_{r}\cdot T_{c}/n\right)$;
$\beta_{r}$ is the ideal front-end complex bandwidth (with a brick-wall filter (Hz)).

In Equation (12), the term in square brackets reflects the squaring losses attributed to the non-coherent discriminator computations, while the term in braces results from approximations depending on the value of Δ⋅b. Given a fix front-end bandwidth and an equivalent chip spacing during tracking, the approximation mainly involves the signal modulation represented by $T_{c}/n$. The Cramer-Rao Lower band is reported by Betz *et al.* [40] to be:

(13) στLB2≅{BL(1−0.5BLTP)2CN0b2,b≤1BL(1−0.5BLTP)2CN0b,b>1

**Figure 8:**
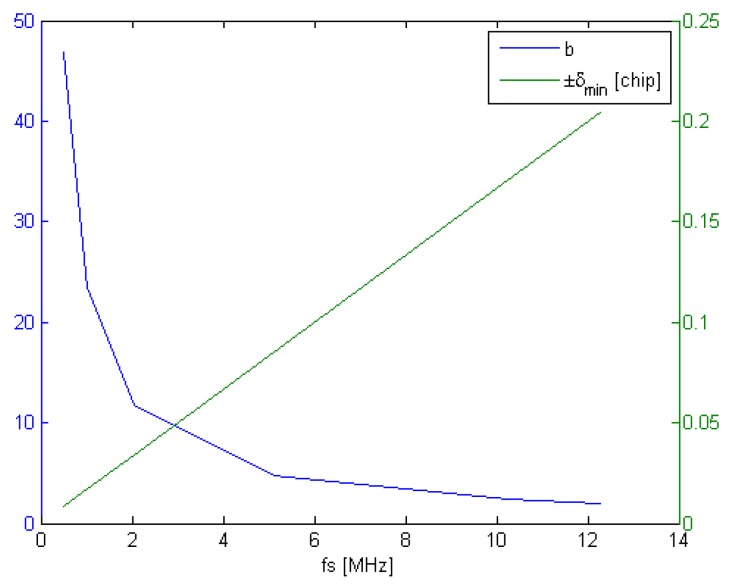
*b* and $\delta_{min}$ *vs.* $1/T_{s}$ with a 22.3 MHz Front-end Bandwidth at 60 MHz.

It thus becomes interesting to determine what DLL noise variance can be expected for each GNSS signal when tracked with the proposed channel. Analysis in [40] reports that for limited front-end bandwidths, the discriminator gain diminishes as the early-late correlator spacing Δ decreases, while increasing the loop bandwidth and thus the loop variance. Three discriminator regions are identified as: Spacing-Limited, Transition and Bandwidth-Limited, in accordance with Equation (12). Looking at MBOC, while assuming $\beta_{r}=22.3$ MHz and $T_{c}/n=1/\left(12\cdot 1.023\times 10^{6}\right)$, b≈2 for the BOC(6,1) signal component, which rapidly falls under the Bandwidth-Limited during tracking area with Δ≤0.5 chip. One should bear in mind that the relative power ratio of BOC(6,1) is one tenth that of BOC(1,1), for which b≈12, well within the Spacing-Limiting function. Looking at other GNSS signals, it appears that in the presented configuration, b ranges from 2 to 47, as depicted in Figure 8. For signals where *b* is high, it still is beneficial to reduce δ, also mitigating multipath errors. Nevertheless, unless dedicated RAM blocks are available for all the code phases used, a 60 MHz sampling frequency poses a 1 sample limit on δ based on delayed code phases based on the shift registers approach described above, the impact of which will vary with the GNSS signal chipping rates.

#### 2.1.7. Power Consumption and Resource Usage

Table 3 summarizes both the power consumption, as obtained with the Xilinx ISE XPower software, and the FPGA resource usage for different tracking channel complexities, leading to the proposed universal channel. The power consumption percentages presented herein are taken relatively to the “BPSK with FDMA” reference implementation, assessing the overhead associated with the implementations derived with added feature sets.

It can be seen that the quiescent power is relatively constant across all implementations, and may be attributed to the chip itself, leaving the dynamic power as a more meaningful comparison metric. The single-component MBOC implementation consumes 33% more power, while the dual-component (data and pilot) requires twice as much, *i.e.*, 66% increase compared to the reference BPSK implementation.

For each implementation, the absolute number of resources and associated percentage (*vs.* available) are presented. As a result, the proposed optimizations led to a dual-component MBOC universal channel of complexity comparable to that of two traditional BPSK reference channels, but with a lot more flexibility. For flexibility and maintainability, the universal channel has been implemented with VHDL configurations that can easy be changed to enable or not several feature sets.

### 2.2. Universal GNSS Channel Validation

**Figure 9:**
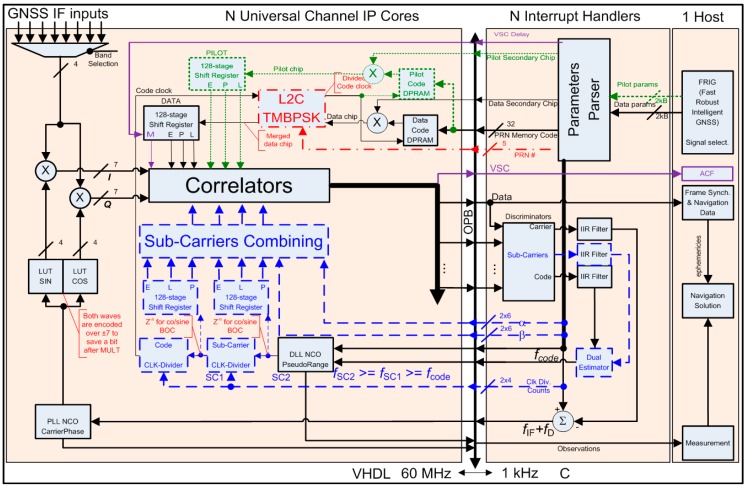
Proposed Universal Channel High Level Architecture.

The resulting architecture of the proposed GNSS Universal Channel is presented in Figure 9, where different colors help highlight added feature sets:

- red dotted and dashed lines for L2C TMBPSK of a L2CM memory code with a locally generated L2CL code,
- blue dashed lines for MBOC sub-carriers replicas generation and feedback,
- green dotted lines for dual-component overhead (extra correlators and sub-carriers combining not shown),
- purple solid lines for the optional Variable Spacing Correlator (VSC) used to plot Auto-Correlation Function (ACF) plots.

The following sections present the different test scenarios conducted to validate the proposed architecture in terms of constellations and signals on different frequency bands, with different spreading codes and modulations. The reader should be advised that this paper focuses on available civil signals, although its architecture also applies to restricted access signals.

#### 2.2.1. Constellation Compatibility

As summarized in Table 1, the four GNSS constellations provide similar (for GNSS compatibility), yet distinctive (for GNSS interoperability) modulation characteristics. On top of that, most signals have their own navigation message definition, including preamble synchronization, parity checking, framing, interleaving and encoding, all of which are defined in their respective Interface Control Documents (ICD), or Interface Specification (IS) [10,11,41,42,43,44]. All four constellations have their own geodetic and timing systems, but provide (now or in a near future) information to relate with other GNSS. Such navigation message data fusion into a common solution add to the complexity of a universal receiver is outside the scope of this paper, rather focused on signal processing. In order to demonstrate the proposed universal channel compatibility with all constellations, at least one signal of each is acquired and tracked.

#### 2.2.2. Frequency Bands Compatibility

Although this is not a feature related to the universal channel per se, being able to acquire and track signals on all bands has a net advantage in terms of both frequency diversity for improved ionosphere error correction in an autonomous receiver, as well as enhanced resistance to interference. In the current implementation, a super-heterodyne RF front-end approach is used, where a configurable Local Oscillator (LO) takes the Radio-Frequency (RF) signal down to 70 MHz, which is then processed by a 24 MHz wide band-pass filter and down-converted to IF with a common 55 MHz LO. All LOs and clock are synchronized through an external 10 MHz reference clock.

#### 2.2.3. Spreading Code Schemes Compatibility

Spreading codes are probably the greatest source of variation among all signals. Indeed, different types are currently broadcast: Gold, Weil, Maximal Length, short cycled linear patterns, *etc.* Rather than deploying dedicated logic to support all signals, a universal memory code approach is used. The remaining signal-specific configuration parameters are the code length and its chipping rate; the only exception being the GPS L2CL code generated with LFSR logic.

#### 2.2.4. Modulations Compatibility

For compatibility sake, GNSS signals are based on a few modulation types, all derived from PSK and BOC.

#### 2.2.5. GNSS Test Scenarios

To cover all the above signal particularities and to demonstrate the proposed universal channel, the resulting test scenarios are summarized in Table 4.

One should bear in mind that GPS L2CM is first acquired without L2CL. Because of their continuous time multiplexing, the L2CM spreading code (*i.e.*, transmitted at 511.5 kchip/s) correlator spacing is limited to ±0.24 chip in order to avoid being polluted by L2CL. Also, with GLONASS being transmitted at 511 kchip/s, the 60 Msample/s channel design does not allow for a correlator spacing greater than ±31 samples, *i.e.*, ±0.26 chip. On the other hand, GPS L5 at 10.23 Mchip/s suffers from the opposite problem: the channel sampling rate cannot achieve better than ±1 sample, *i.e.*, ±0.17 chip.

Unfortunately, cBOC modulation could not be formally tested without the publically undisclosed Galileo E1A and E6 spreading codes. The same applies to modernized signals that are not yet available in space, such as GPS L1C TMBOC. The test scenarios are further described in the following paragraphs.

##### Galileo E1 B&C

CBOC is an implementation of the MBOC spectrum, involving the two (6× and 1×) sub-carriers as in GPS L1Cp, but with different, yet constant amplitudes. The current implementation being based on integrations over multiples of 1 ms, a total of four partial integrations are accumulated (coherently or not) to match the full 4 ms long primary spreading code before proceeding to the next cell of the acquisition span, encompassing 4092 chips and 16 Doppler 667 Hz bins.

During acquisition, only the BOC(1,1) sub-carrier is used, with a ±28 samples correlator spacing. This early simplification can be used since the second sub-carrier only bears a tenth of the signal power, which can be neglected. Once synchronized with a BOC(1,1) modulation, the full CBOC replica signal may be generated locally with a reduced correlator spacing of ±3 samples.

The 12-bit un-encoded preamble may be used to synchronize onto the message frame of the data component. On the other hand, the pilot component bears a 25-bit secondary code.

##### GPS L1C

GPS L1C modernized signal involves BOC on the data component and TMBOC on the pilot. Although BOC(1,1) provides a similar effect than the Manchester code, TMBOC requires further thoughts. In the specific case of GPS L1Cp, BOC(1,1) and BOC(6,1) are alternatively enabled over a pre-determined 33-chip long sequence; 33 being an integer factor of the 10,230 chip long primary code length. Considering a dual-component channel (processing a total of four sub-carriers), at most 4096 chips can be saved in a 16 kbit RAM. This is insufficient to match the longest primary codes. Hence, a shorten pattern is repeatedly applied based on lower bits of the primary chip address bus, *i.e.*, modulo the shorter length. This memory is thus written via a 32-bit data bus, but read four bits at a time. A minimal set is pre-computed prior being repeated to fill out the RAM for future compliance (with the modulo operator %):

(14) $$\frac{33\;\text{chips}\;\times 4\;\text{sub}-\text{carriers}\times 8\;\text{repeats}}{32\;\text{bits}}=33\;\text{memory addresses of pre}-\text{computed pattern}$$

(15) $${\text{addr}}_{\text{TMBOC}}={\text{addr}}_{\text{primary code}}\%33$$

The resulting enable bits are applied at step 3 of the acquisition process, defined in Section 2.1.2. Other signals are always enabled; the memory being filled with ‘1’.

In terms of the navigation message, CNAV-2 [11] requires a different approach than for CNAV used in both GPS L2C and L5. In fact, synchronization can be achieved on the Bose, Chaudhuri, and Hocquenghem (BCH) encoded 52-symbol Time of Interval (TOI), provided the receiver knows what to expect for the next frame. This prerequisite knowledge can be extrapolated from other signals tracked from the same broadcasted satellite. This approach is preferred over looking for “non-variable” data from sub-frame 2 that is both encoded with Low Density Parity Check (LDPC), and interleaved with sub-frame 3 using a 38 rows and 46 columns matrix.

Since GPS block III satellites (*i.e.*, the first intended to broadcast the modernized GPS L1C signal) scheduled for 2014 [45] have not been launched yet, chronograms were used to show sub-carrier weights in time for the pilot component.

##### GPS L2C

In the case of GPS L2C, the 10,230 chip-long L2CM code is to be generated in (nominally) 20 ms, *i.e.*, 511.5 chips per ms. To avoid partial chip every other ms, a 2 ms coherent integration approach is used. During acquisition (solely based on L2CM), 10 such 1023 chip long partial correlations are required to parse the full L2CM code. This acquisition is performed with a correlator spacing of ±28 samples (based on 60 Msample/s), avoiding any effect from the L2CL spreading code.

The resulting search span, involving 20 ms iterations being repeated over the 10,230 different chip alignments and the 31 Doppler bins (each separated by 2/3×Tc=333 Hz), could reach up to 6342.6 s, *i.e.*, almost 2 h. To avoid this unacceptable worst case unaided sequential acquisition time, the satellite L1 C/A signal information can be extrapolated, provided its prior acquisition ( the navigation bit transition being aligned with the L2CM code start), leading to the following set of Equations:

(16) $${\text{Doppler}}_{L2C}=\frac{L2}{L1}\cdot {\text{Doppler}}_{L1\;C/A}$$

(17) $$\lfloor \frac{c_{L1\;C/A}}{2}\rfloor =c_{L1CM}\%1\;\text{ms}$$

(18) $$c_{L2CM}=c_{L2CL}\%\;20\;\text{ms}$$

(19) $$c_{L1\;C/A}=c_{L2CM}=0\;\text{whenever a}\;L1\;C/A\;\text{navigation bit transition occurs}$$

(20) $$c_{L1\;C/A}=c_{L2CM}=c_{L2CL}=0\;\text{whenever a}\;L1\;C/A\;\text{or}\;L2C\;\text{navigation frame starts}$$

In fact, the universal channels are synchronized with a global 1 ms pulse, allowing for a triggering mechanism to initialize the code generation at any given chip, at a given time stamp. Equation (20) is a simplification as the 1.5 s L2CL code period starts more often than at the 6 s NAV or 12 s CNAV frames.

The CNAV navigation symbols being transmitted at 50 symbol/s, a full L2CM period must be accumulated to obtain one symbol. In order to synchronize onto the frame and to overcome its Forward Error Correction (FEC) encoding with a ½ ratio, a pattern composed of the common 8-bit preamble followed by the satellite-specific 6-bit PRN is used: Of the resulting 28 encoded symbols, the last 16 are not affected by the unknown data from the previously broadcasted frame [44]. These are then used to locate the beginning of a frame at an offset of 12 symbols. Once, the navigation data is obtained, the L2CL offset can be assessed prior to merging it with the L2CM stream, achieving a 3 dB gain with a TMBPSK match filter approach. This multiplexing requires a clock with twice the rate, *i.e.*, 1.023 Mchip/s, which is then divided down to 511.5 kchip/s for the codes generation. The same 2 ms integration time is preserved in order to keep integrating over an integer number of chips for each of L2CM and L2CL codes.

##### GPS L5

GPS L5 shares the same CNAV navigation data than L2C, although it is broadcasted twice as fast, allowing for the same frame synchronization scheme to be applied [41]. GPS L5 transmits 10,230 chips every 1 ms period; the chipping rate must be 10 times faster than for GPS L1 C/A, *i.e.*, 10.23 Mchip/s. Considering a 60 MHz sampling frequency, the acquisition is performed with a ±3 samples correlator spacing.

##### GLONASS L1OF

The RF front-end must support the 1602 MHz frequency, while the IF to baseband frequency down-conversion stage must also support the several FDMA channels, allowing them to be seamlessly tracked, independently from their different frequency offset. In fact, this frequency offset is pre-determined and associated with each PRN, relieving the universal channel from this signal type management.

Because of HW design limitations, a correlator spacing of ±31 samples is used during acquisition, which roughly corresponds to ±¼ chip. In cold acquisition, because two satellites share the same RF offset, the navigation data must be decoded (no encryption) to corroborate that the expected satellite is effectively being tracked. This information is not available in every 2 s long string, and may thus require longer decoding to find out.

##### BeiDou B1-I

This last signal, from the BeiDou Phase II constellation requires an RF front-end capable of processing 1561.098 MHz, with a 2046 Mchip/s. The initial correlator spacing is set to ±14 samples during acquisition. The 11-bit un-encoded preamble may be used to synchronize onto the message frame, once the 10-bit secondary code has been wiped out.

## 3. Results

The proposed dual-component universal channel ends up using twice the resources of a traditional GPS L1 C/A tracking channel, in exchange for the flexibility of tracking any GNSS signal (including both pilot and data, whenever applicable). Furthermore, it has a low worst-case 42 mW/channel dynamic power consumption. This corresponds to a 66% increase compared to the reference BPSK single-component channel. Keeping in mind that a FPGA, such as the one used in the current implementation, reaches consumption as much as 12 times that of a comparable size Application Specific Integrated Circuit (ASIC) [46], an equivalent ASIC implementation power consumption would 3.5 mW/channel, or 2.1 mW/channel for the simplest BPSK implementation. These values are comparable to the consumption of the u-blox GPS L1 C/A with SBAS receiver chip specified to be 67 mW for 50 channels (1.34 mW/channel) [47].

**Figure 10:**
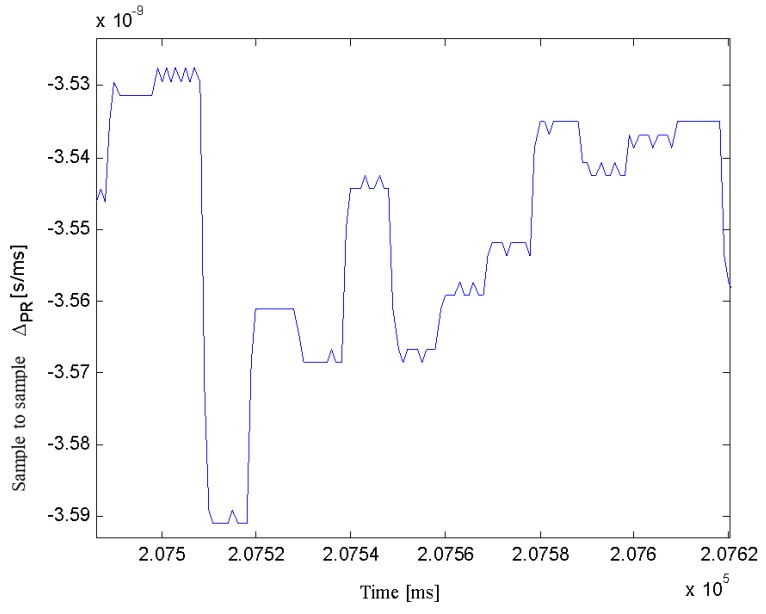
GPS L1 C/A LoS Variation (s) Observed every 1 ms Epoch, Based on 10 ms Non-Coherent Integration Time DLL Feedback Commands.

Furthermore, the current processing bottleneck is the discriminators computation through the 1 kHz interrupt sub-routine/channel in the embedded MicroBlaze 5.0 processor, which could be resolved with a newer, more powerful, chip. Alternatively, the navigation message decoding could be performed in an external processor. Note that in all cases, except for GPS L2C, a 10 non- coherent integrations of 1 ms is performed prior to computing the DLL feedback. The resulting commands lead to a trend represented in Figure 10, where Low Significant Bit (LSB) oscillations may be observed between commands reaching up to $0.06\;\times 10^{-9}$ s/ms. These equivalent 18 m/ms jumps are smoothed out in the PVT solution, computed at up to 100 Hz.

Another performance assessment is the instantaneous channel update mean time of 1.07 ms, allowing the receiver to rapidly adapt to its sensed environment. Finally, the 530+ civil signal components occupy a total memory codes size of ~5 MB (exception made of the L2CL code). Hence, pre-computed spreading codes may easily be stored in external memory and used on demand.

A metric to consider when seeking for the best signals to track is their pseudo-range noise. The pseudo-range being proportional to the propagation time (*cf.* Equation (21)), it should behave approximately as a parabola for a static observer, from horizon to zenith and horizon again. Its second derivative should thus tend towards a constant value. The DLL feedback can be approximated as the first derivative of the propagation time (*cf.* Equation (22)), as shown in Figure 10.

The pseudo-range noise—a random process with an order greater than 2—may then be approximated as the remaining variations of the second derivative of the pseudo-range (*cf.* Equation (23)) [48], which is generalized to a partial code within 1 ms (in Equation (24)) for the particular case for L2C (outlined in Equation (25)). At that level, only the chip index and the phase of the chipping rate clock signal, taken at 1 kHz, need to be considered ([49] (p. 264). This simplification is useful in analyzing signals, as the associated navigation message does not need to be accounted for. Moreover, multi-frequency signals being characterized by different paths, the extra time offset may then be neglected [50]. Noise is then quantified as the standard deviation of the second derivative of the pseudo-range $\sigma_{\eta_{PR}}$:

(21) $$T_{prop.}=N_{code}\cdot T_{code}+\frac{N_{chip}+\Theta_{chip}}{f_{chip}}$$

(22) $$\varepsilon_{PR}\propto {\dot{T}}_{prop.}=\frac{\frac{\partial }{\partial t}\left\{N_{chip}+\Theta_{chip}\right\}}{f_{chip}}\left[\frac{s}{s}\right]$$

(23) $$\eta_{PR}\propto {\ddot{T}}_{prop.}=\frac{\frac{\partial^{2}}{\partial t^{2}}\left\{N_{chip}+\Theta_{chip}\right\}}{f_{chip}}\left[\frac{s}{s^{2}}\right]$$

(24) $${\eta_{PR}|}_{\text{partial code}}\propto \frac{\frac{\partial^{2}}{\partial t^{2}}\left\{{N_{chip}|}_{1\;\text{ms}}\%\left(f_{chip}\cdot 1\;\text{ms}\right)+{\Theta_{chip}|}_{1\;\text{ms}}\right\}}{f_{chip}}\left[\frac{s}{s^{2}}\right]$$

(25) $${\eta_{PR}|}_{L2CM}\propto \frac{\frac{\partial^{2}}{\partial t^{2}}\left\{{N_{chip}|}_{2\;\text{ms}}\%1023+{\Theta_{chip}|}_{2\;\text{ms}}\right\}}{511,500}\left[\frac{s}{s^{2}}\right]$$

where:

$T_{prop.}$ is the propagation time;
$N_{code}$ is the number of complete code;
$T_{code}$ is a complete code period;
$N_{chip}$ is the chip index of the primary code;
$\Theta_{chip}$ is the phase of the chipping rate clock;
$f_{chip}$ is the chipping rate;
$\eta_{PR}$ is the pseudo-range noise.

**Figure 11:**
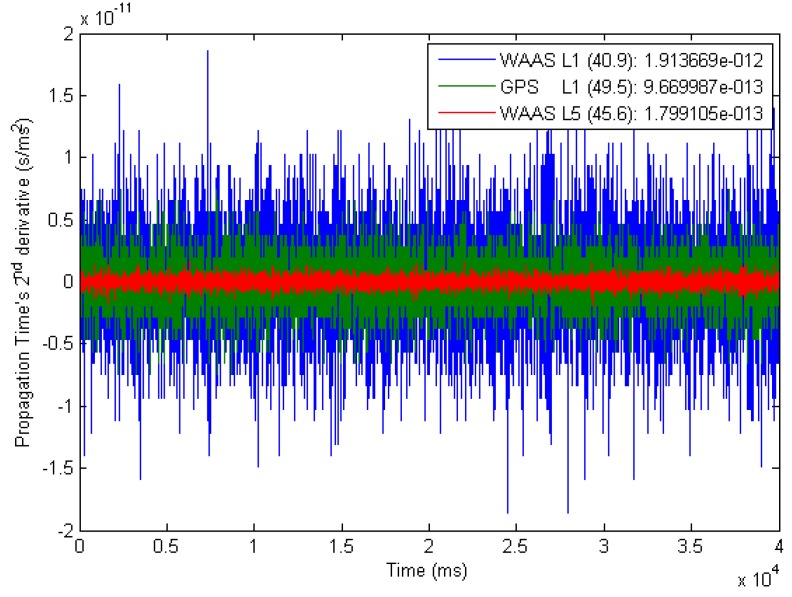
Propagation Time Noise During 40 sLegend: (signal type) $\left(\langle C/N_{0}\rangle \right)$ : (pseudo-range noise standard deviation).

Such a snapshot analysis is displayed in Figure 11, where the legend indicates the signal type, its average $C/N_{0}$ and its pseudo-range noise standard deviation $\sigma_{\eta_{PR}}$).

**Figure 12:**
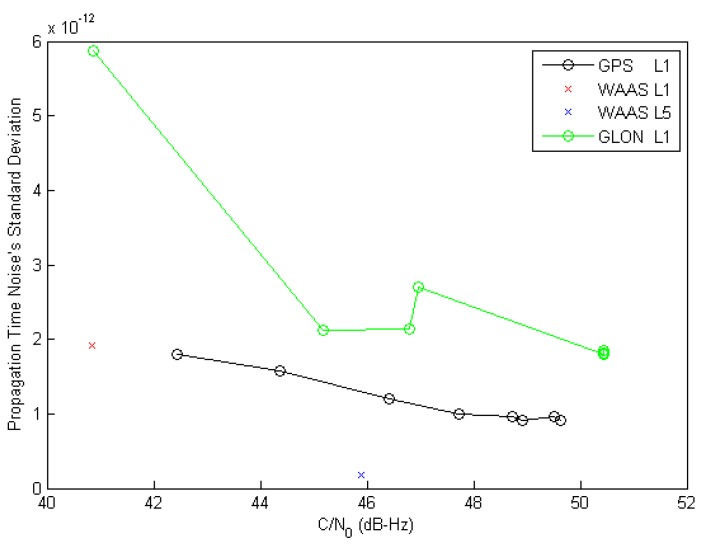
Different Correlator Spacing (Through Different Signals) Impact on DLL Noise *vs.* $C/N_{0}$.

Different signals pseudo-range noise is further compared *vs.* signal strength in Figure 12, where it can be seen that WAAS L1 quality is in line with that of GPS L1 C/A (as they have the same chipping rate), while GPS L5 appears 10 times better for a given C/N_0_ (with 10 times the chipping rate). Signals intrinsic phase noise can be a dominant contributor to carrier and pseudo-range measurement performance, resulting in measurement errors.

**Figure 13:**
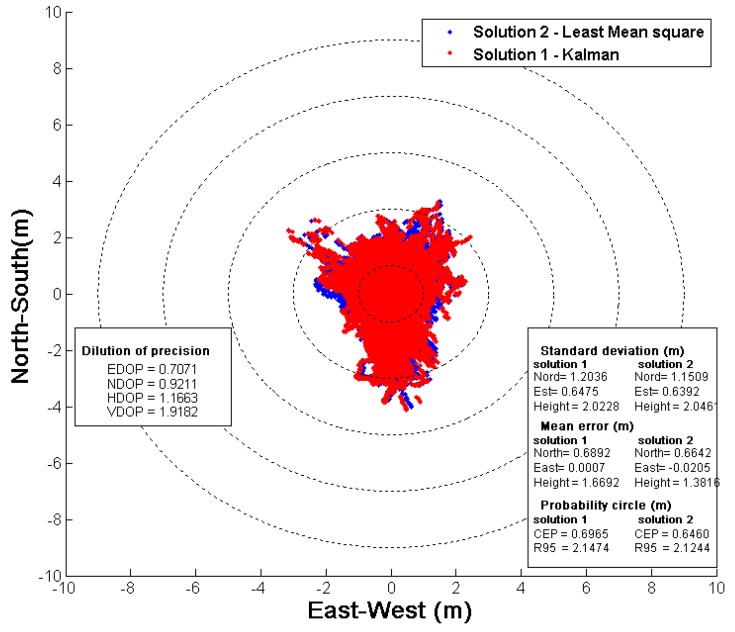
Relative 2D error of a GPS L1 C/A WAAS Augmented Solution.

In Figure 13, a 5 Hz static GPS L1 C/A WAAS augmented solution is presented with a 15° elevation mask with 4-bit quantization. Note that the pre-computed 0-baseline reference position used has a 2 mm (95%) error.

## 4. Conclusions

It has been shown that with their longer codes modernized signals take longer to acquire. In order to minimize that impact, a preliminary solution based on GPS L1 C/A signals can be leveraged to reduce the search span of other signals from tracked satellites. This transition from old to new signals of any given satellite, emphasizes the need for a universal channel approach, avoiding many idle dedicated-channels. This power reduction strategy is especially critical for portable devices whose battery optimization is the challenge of the century. Moreover, a commercial application based on such a universal channel could easily introduce a pricing scheme based on available constellation and signal types. Its future compatibility would thus make it a great option for expandable design based on SW only upgrades, easily deployable into already released products.

In this paper, the GNSS signals characteristics have been identified and addressed by a universal acquisition and tracking channel, while maintaining power consumption as low as possible (by avoiding idle channels and sharing resources) and maintaining a high level of robust tracking and flexibility. As shown, the proposed architecture allows sequential acquisition and tracking of any chipping rate, carrier frequency, FDMA channel, modulation—*i.e.*, BPSK(q), QPSK(q), sin/cos BOC(p,q), $\text{CBOC}\left(r,\;p,\;P_{r},\;\pm \right)$ and $\text{TMBOC}\left(r,\;p,\;w_{r}\right)$—or constellation, and is totally configurable (any integration time, discriminator, *etc.*) within a fast turn-around time.

Also, its dual-component architecture allows for two sequential acquisition options: (1) dual secondary chip estimation and (2) dual primary code delay (twice as fast) estimation for a single-component acquisition. Moreover, its upgradable memory codes and sub-carriers configurability (co/sine phase and α and β weights in time) make it future-compliant for any variation that could be proposed for GLONASS modernization and BeiDou phase III signals, the final description of which are yet to come.

These benefits came at the cost of increasing the tracking channel correlators number from six (NEML reference BPSK architecture) to eight for a single-component (or equivalently 16 for a dual-component) GNSS channel, thanks to the proposed resources reduction. All these GNSS signal tracking features result in a 66% power consumption increase compared to those used in a reference BPSK channel. Nevertheless, a dual-component channel requires twice the resources of a reference BPSK channel, but with twice the throughput, thus achieving an equivalent resource per channel ratio. Also, the proposed TMBOC combined with the 2 phase-controlled sub-carriers approach could be reused to implement the AltBOC sub-carrier weights with a periodicity of eight sub-chips, provided the RF front-end and sampling frequency could process a bandwidth of least 51 MHz.

### Opening on Satellite Selection

The satellite selection problem has been addressed through many different approaches, leading to computationally (sub-)optimized algorithms [51]. Nevertheless, in the context of a universal channel, the challenge becomes selecting the next best signal, not only the next best satellite. Not only the number of option increases, but also does the selection algorithm complexity, thus requiring a re-spin of the existing selection algorithms. Indeed, the satellites geometry may not be sufficient anymore: frequency diversity targeting ionosphere corrections, signal effective (Gabor) bandwidth for precise pseudo-ranging measurements, signal availability in case of jamming as well as measurement ambiguity are all contributing factors to be accounted for, *i.e.*, a new research topic in itself that could address the traditional satellite navigation limitations at once.

Some thoughts on such a signal selection strategy go as follows. On cold start, all GPS L1 C/A signals are searched for in parallel to reduce as much as possible the Time To First Fix (TTFF). Once ephemerides are downloaded, known visible satellite orbits allow channels to be progressively assigned to modernized signals (most of which have dual-components, as seen in Table 1) from the same constellation, eventually on different frequency bands if the RF front-end allows, with a reduced search grid (*i.e.*, known Doppler and estimated chip alignment). Furthermore, this initial Position Velocity and Time (PVT) solution would help reducing the search span of signals on other constellations. A key factor in resolving this signal selection challenge consists in maintaining the ideal pool of next best signals based on an adapted version of the FRIG algorithm [51]. Hence, the proposed universal channel combined with a new signal selection algorithm and a navigation data fusion strategy could become a powerful tool in cognitive receivers.

## Figures and Tables

**Table 1:** GNSS Signals Survey.

System	# SV	Center Freq. (MHz)	Broadcast BW (MHz)	Signal Component	Modulation Type (*f_r_ = 1023* kHz)		Phase (°)	Gabor (MHz)	Code Length (chip)		Code Period (ms)		MTTA (s)		Symbol Rate (symbol/s)	Data ambiguity	Forward Error Correction	Earth Power (dBW)
									Primary	Secondary	Primary	Secondary	Primary	Secondary				
**GPS / WAAS**	32/24 + 3/3	L1: 1575.42	24	L1C/A	BPSK(1)		90	2.046	1023	0	1	0	16	0	50	20	none	−158.50
				L1P(Y)	BPSK(10)		0	20.460	6.187E+12	0	6.05E+08	0	3.4E+28	0	50	0	none	−161.50
				L1M	sBOC(10,5)		90	30.690	undisclosed		undisclosed						FEC(½)	−158.00
				L1C-I	sBOC(1,1)		0	4.092	10,230	0	10	0	15634	0	100	1	BCH/LDPC (½)	−163.00
				L1C-Q	TMsBOC(6,1,4/33)		0	14.322	10,230	1800	10	18000	15634	4.427E+09	---	---	---	−158.25
				WAAS-L1	BPSK(1)		90	2.046	1023	0	1	0	16	0	500	2	171o; +133o	−161.00
		L2: 1227.60	24	L2CM	BPSK(½)	TMBPSK (½,½)	90	2.046	10,230	0	20	0	62328	0	50	1	171o; +133o	−160.00
				L2CL	BPSK(½)		90		767,250	0	1500	0	2.6E+10	0	---	---	---	
				L2P(Y)	BPSK(10)		0	20.460	6.187E+12	0	6.05E+08	0	3E+28	0	50	0	none	−161.50
				L2M	sBOC(10,5)		90	30.690	undisclosed		undisclosed						FEC(½)	−158.00
		L5: 1176.45	24	L5-I	QPSK(10)		0	20.460	10,230	10	1	10	155	8	100	1	171o; +133o	−157.90
				L5-Q			90	20.460	10,230	20	1	20	155	61	---	---	---	−157.90
				WAAS-L5	BPSK(10)		0	20.460	10,230	0	1	0	155	0	500	1	171o; +133o	−154.00
**Galileo / EGNOS**	(2+4+2)/30 + 3/3	L1: 1575.42	40.92	E1B	CsBOC(6,1,1/11,+)		0	14.322	4092	0	4	0	1010	0	250	1	171o; −133o	−160.00
				E1C	CsBOC(6,1,1/11,−)		180	14.322	4092	25	4	100	1010	1899	---	---	---	−160.00
				EGNOS	BPSK(1)		90	2.046	1023	0	1	0	16	0	500	2	171o; +133o	−161.00
				E1A	cBOC(15,2.5)			35.805	undisclosed		undisclosed				100			
		E6: 1278.75	40.92	E6A	cBOC(10,5)			30.690	undisclosed		undisclosed				100			
				E6B	BPSK(5)		0	10.230	5115	0	1	0	78	0	1000	1	171o; −133o	−158.00
				E6C	BPSK(5)		180	10.230	5115	100	1	100	78	7596	---	---	---	−158.00
		E5a:1176.45	20.46	E5a-I	QPSK(10)	AltBOC(15,10)	0	20.460	10,230	20	1	20	155	61	50	1	171o; −133o	−158.00
				E5a-Q			90	20.460	10,230	100	1	100	155	7596	---	---	---	−158.00
		E5: 1191.795	92.07				8-PSK	51.150					0	0				−152.00
		E5b:1207.14	20.46	E5b-I	QPSK(10)		0	20.460	10,230	4	1	4	155	0	250	1	171o; −133o	−158.00
				E5b-Q			90	20.460	10,230	100	1	100	155	7596	---	---	---	−158.00
**GLONASS / SDCM**	28/24 + 2/3	L1g: 1602.00	17.5275	L1OF	BPSK(~½) - FDMA		90	8.335	511	0	1	0	8	0	100	10	none	−161.00
				L1SF	BPSK(~5) - FDMA		0	17.533	undisclosed						50			
		L1: 1575.42		L1OC	BOC(n,n)		0		undisclosed									
				L1SC					undisclosed									
				SDCM					undisclosed									−158.00
		L2g: 1246.00	15.9075	L2OF	BPSK(~½) - FDMA		90	6.710	511	0	1	0	8	0	100	10	none	−167.00
				L2SF	BPSK(~5) - FDMA		0	15.908	undisclosed						250			
		L2: 1227.60		L2OC				13.683	undisclosed									
				L2SC				13.683	undisclosed									
		L3: 1202.025		L3OC-D	QPSK(x)		0	20.460	10,230	5	1	5	155	1	200	1	171o; +133o	
				L3OC-P			90	20.460	10,230	10	1	10	155	8	---		---	
		L3: 1208.088		L3SC									0	0				
		L5: 1176.45		L5OC			0	16.368	undisclosed									
**BeiDou / SNAS**	(5/27 + 5/3 + 6/5) + 1/3	B1-1: 1561.098	4.092	B1-I: C/A	QPSK(2)	AltBOC(14,2)	0	2.046	2046	20	1	20	31	61	50	1		−163.00
				B1-Q: military			90	2.046			>400							
		L1: 1575.42						2.046	2046	0	1	0	31	0	500	2		−163.00
		B1-2: 1589.74	4.092	B1-2: military	QPSK(2)		0	2.046	filed at ITU, although nothing is being broadcast									
							90	2.046										
		E5b:1207.14	20.46	B2-I: C/A	BPSK(2)		0	2.046	2046	20	1	20	31	61	50	1		
				B2-Q: military	BPSK(10)			20.460			>160							
		B3: 1268.52	20.46	B3-I: C/A	QPSK(10)		0	20.460	10,230	20	1	20	155	61	50	1		
				B3-Q: military			90	20.460			>160							
		L1: 1575.42		B1-Cd	MBOC(6,1,1/11)			14.322			OS				100		½	
				B1-Cp											---			
				B1d	sBOC(14,2)			32.736			AS				100		½	
				B1p											---			
		B2: 1191.795		B2ad	QPSK(10)	AltBOC(15,10)	0	20.460							50		½	
				B2ap			90	20.460							---			
							8-PSK	51.150			OS							
				B2bd	QPSK(10)		0	20.460							100		½	
				B2bp			90	20.460							---			
		B3: 1268.52		B3	QPSK(10)		0	20.460			AS				500		none	
							90											
				B3-Ad	sBOC(15,2.5)			35.805			AS				100		½	
				B3-Ap	sBOC(15,2.5)										---			

**Table 2:** BOC Tracking Channel Architectures Classification.

Category	Main Methods	Approach
Narrow Correlators	Double-Delta (DD) [14]; High Resolution Correlator (HRC) [15]; BOC universel [16]	Narrow tracking once aligned with the main peak of the correlation curve. Weak performances in presence of noise and multipath. A complex combination of absolute values of correlators approaches the BPSK triangular ACF shape during initial alignment.
Single Lobe (~BPSK)	Single Side Lobe (SSL) [17]; Dual Sideband (DS) [18]	Independently process main lobe(s), achieving BPSK-like correlation curve.
Extra-Correlators	Bump and Jump (BJ) aka Very Early Very Late (VE-VL) [19]	Extra correlators allow monitoring secondary peaks of the correlation curve. Their location depends on the targeted modulation scheme.
Replica Spreading Code Combination	Time-Multiplexed BOC(6,1) (TM61(a)) [20]; Shaping Correlator Receiver (SCR) [21]; S-Curve Shaping [22]; Code Composite Ranging Waveform (CCRW) [23]; Strobe Correlator [24]; Autocorrelation Side-Peak Cancellation Technique (ASPeCT) [9]	Minimize secondary peaks of the correlation curve by combining different spreading codes into the local replicate signal. Despite good multipath performances, these approaches suffer from higher noise levels as they are not based on the Maximum Likelihood “Matched Filter”-like Correlator, targeting the Cramer-Rao lower bound. Furthermore, S-Curve Shaping would require a minimum sampling frequency reaching 200 MHz to track MBOC signals. ASPeCT only applies to BOC(p,p).
Extra-Loops	Sub Carrier Phase Cancellation (SCPC) [17]; Triple-Loop Dual-Estimator (TLDE) [25]	Tracking of sub-carrier on top of carrier and code, avoiding periodic signal integer uncertainty.
Frequency Response	Channel Transfer Function H(f) [26]; Symmetric Phase-Only Matched Filter (SPOMF) [27]	Frequency-domain analysis is more flexible and precise, no matter what the signal modulation is; at the extra cost (e.g., hardware resources) of direct and inverse Fourier transforms.
Loop filters	A shared Extended Kalman Filter (EKF) is used to compute the channels feedback [28,29]. VDLL could also be considered to distinguish main peak from secondary ones by eliminating solutions with larger positioning residues. EKF can also be used as individual tracking channel loop filter [30]	Vectorial DLL (VDLL) allows for inter-channel assistance, minimizing satellite loss and reacquisition occurrences by replacing independent loop filters by integrated EKF. Was successfully applied to BPSK tracking. Independent Extended Kalman Filtering (EKF) could also be used to compute the loop feedback in every channel. Both approaches could be adapted to BOC.
Time-Domain Analysis	Vision correlator [31]	Extra complex integrator measurements are taken at slightly different time offsets in order to assess the chip transition in the time-domain. This method could be applied to sub-carrier transitions as well.
Signal assistance	Combined Signals [30]	GPS L1 C/A combined with GPS L1C for enhanced tracking

**Table 3:** Universal Channel Resources for Different Feature Sets.

Resources in xc4vsx55-10ff1148	Available	BPSK w/FDMA		L2C		BOC		Single MBOC		Dual MBOC	
Dynamic Power (mW)		25.1	100%	25.1	100%	29.6	118%	33.4	133%	41.8	166%
Quiescent Power (mW)		860	100%	860	100%	860	100%	861	100%	861	100%
Total Power (mW)		885	100%	885	100%	890	101%	894	101%	903	102%
Slices	24576	651	2.6%	765	3.1%	943	3.8%	1018	4.1%	1410	5.7%
Slice Flip Flops	49152	775	1.6%	918	1.9%	1118	2.3%	1186	2.4%	1476	3.0%
4 input LUTs	49152	908	1.8%	1127	2.3%	1456	3.0%	1554	3.2%	2123	4.3%
as logic		894		1113		1436		1528		2091	
as shift registers		14		14		20		26		32	
FIFO16/RAMB16s	320	2	0.6%	2	0.6%	2	0.6%	3	0.9%	4	1.3%
DSP48s	512	11	2.1%	11	2.1%	17	3.3%	17	3.3%	29	5.7%
Max. number of single channels			37		32		26		24		17
Max. number of dual channels			18		16		13		12		17

Legend: Dynamic Power identifies 66% power consumption increase of dual MBOC compared to BPSK feature set. Each Feature Set column and Max. number of channels row are color scaled to highlight best to worst.

**Table 4:** Proposed Universal Channel Test Scenarios.

	GNSS	RF (MHz)	Signal	Primary Code	Modulation	±δ (Chip)	Particularity
1	GPS	1575.42	L1C	10 ms; 10,230 chips	I: sBOC(1,1); Q: TM-sBOC (6, 1, 4/33)	0.48→0.05; 0.48→0.05	L1 band; GPS; MBOC
2	GPS	1227.60	L2C	L2CM: 20 ms; 10,230 chips; L2CL: 1.5 s; 767,250 chips	TMBPSK (½, ½, ½)	0.24→0.05; off→same as L2CM	L2 band; LFSR logic; TMBPSK
3	GPS	1176.45	L5	1 ms; 10,230 chips	QPSK (10)	0.50→0.17; 0.50→0.17	L5 band; QPSK; Code rate
4	Galileo	1575.42	E1 B&C	4 ms; 4092 chips	CsBOC (6, 1, 1/11, ±)	0.48→0.05; 0.48→0.05	Galileo; MBOC; Code period
5	GLONASS	1602.00	L1OF	1 ms; 511 chips	BPSK(~½)	0.26→0.05	Δ RF in L1; GLONASS; FDMA
6	BeiDou	1561.098	B1-I	1 ms; 2046 chips	BPSK(2)	0.48→0.03	Δ RF in L1; BeiDou; Code length
